# Preoperative Serum Calcitonin Level and Ultrasonographic Characteristics Predict the Risk of Metastatic Medullary Thyroid Carcinoma: Functional Analysis of Calcitonin-Related Genes

**DOI:** 10.1155/2022/9980185

**Published:** 2022-03-02

**Authors:** Yi Fan, Haishan Xu, Meiyan Lv, Ning Li

**Affiliations:** ^1^Department of Ultrasound, The No. 1 People's Hospital of Yongkang, Yongkang, China; ^2^Department of Ultrasound, Sir Run Run Shaw Hospital, Zhejiang University, Hangzhou, China; ^3^Department of Clinical Laboratory, The No. 1 People's Hospital of Yongkang, Yongkang, China; ^4^Department of General Surgery, The No. 1 People's Hospital of Yongkang, Yongkang, China

## Abstract

**Background:**

Early cervical lymph node (LN) metastasis is an important cause of poor survival in patients with medullary thyroid cancer (MTC). This study evaluated whether the preoperative serum calcitonin level in combination with ultrasonographic features of MTC can be used to assess the LN status as well as predict the risk of metastasis in patients with MTC.

**Methods:**

We retrospectively analyzed the clinical data of 95 patients with MTC, and a nomogram model was constructed and validated. Using integrated database analysis of The Cancer Genome Atlas (TCGA) and Genotype-Tissue Expression (GTEx), we mined pathways wherein *CALCA* is involved, identified calcitonin-related genes, and analyzed their functions.

**Results:**

Correlation analysis revealed a significant association between the infiltrating range, diameter, calcification, blood flow, the preoperative serum calcitonin level, and metastasis. The metastasis risk-prediction model showed great accuracy in determining the risk of metastasis in MTC (area under the curve of the receiver operating characteristic [ROC] curve: 0.979 [95% confidence interval 0.946–1.000]). Decision curve analysis (DCA) showed that the model has excellent clinical utilization potential. Significantly, *CALCA*, the mRNA for calcitonin, was highly expressed in thyroid cancer tissues and associated with the cytokine–cytokine receptor and neuroactive ligand-receptor interaction pathways as well as the cell-adhesion molecules. ROC curve indicated that the *CNTFR*, *CD27*, *GDF6*, and *TSLP* genes, which are related to the cytokine–cytokine receptor interaction pathway, could indicate the risk of metastasis in MTC.

**Conclusions:**

The preoperative serum calcitonin level, in combination with ultrasonographic features, can be used to predict the risk of metastasis in patients with MTC and constitute a noninvasive accurate method for preoperative diagnosis of MTC.

## 1. Introduction

Medullary thyroid carcinoma (MTC) is an undifferentiated malignant tumor that arises from the parafollicular cells of the thyroid (C cells) within the diffuse neuroendocrine cell system [1]. MTC, a rapidly progressive malignancy that has a high metastasis rate and poor prognosis, constitutes only 1% to 2% of all thyroid cancers but accounts for 13% of all thyroid cancer-related mortalities [2]. Furthermore, the MTC tumor cells are found insensitive to radioiodine therapy, and postoperative thyrotropin-suppressive therapy with oral levothyroxine tablets is also unhelpful. Therefore, more aggressive radical surgery is essential even in early stage MTC. In principle, MTC surgery should include all thyroid cancer foci and cervical lymphatic metastases, while ensuring an acceptable rate of postoperative complications [3]. A conservative surgery in combination with an unconfirmed diagnosis is a major cause of poor prognosis and tumor recurrence. Therefore, the preoperative identification of MTC-induced metastases is of great clinical importance.

Early cervical lymph node (LN) metastasis is an important cause of high recurrence and low survival in MTC patients [4]. However, by the time MTC progresses to a thyroid nodule, up to 70% of patients would have developed cervical lymphatic metastases, of which 10% would have already developed distant metastases [5]. Preoperative imaging of the neck for LN metastases can directly determine the surgical approach and further influence the prognosis in MTC [6]. Ultrasonography, which is the preferred imaging test for the detection of MTC, has a low sensitivity for the direct detection of MTC lymphatic metastasis [7]. In the past few years, several studies have been conducted to identify the clinical and imaging factors that predict lymphatic metastasis in patients with MTC; however, the results are not consistent [7–10].

Calcitonin, a peptide hormone that is secreted by C cells, is an important specific clinical marker for the diagnosis of MTC [11]. The early diagnosis of lymphatic metastases in MTC based on the preoperative calcitonin level can facilitate timely radical surgery, which is an important factor for improving the prognosis of MTC patients [12]. Computed tomography (CT) can accurately rule out MTC when the serum calcitonin levels are within the normal reference range. Furthermore, dynamic postoperative observation of the serum calcitonin level can crucially help determine the effectiveness of the surgical intervention as well as the risk of tumor recurrence [13, 14].

This study was aimed at assessing the accuracy of radio imaging-based diagnosis of cervical MTC lymphatic metastases depending on the ultrasonographic characteristics of the primary foci of MTC, in combination with the preoperative calcitonin levels, to predict the metastasis risk of MTC patients and to construct a nomogram-based prediction model.

## 2. Materials and Methods

### 2.1. Clinical Data Collection

This study enrolled 95 patients with MTC who underwent surgery at the First People's Hospital of Yongkang from April 2016 to April 2021. The inclusion criteria were as follows: (1) histopathological confirmation of MTC, (2) data on the preoperative serum calcitonin level, and (3) no history of malignancies in other organs. The exclusion criteria were as follows: (1) absence of lymph node dissection, (2) history of thyroid surgery, and/or (3) presence of hyperparathyroidism, infectious disease, and/or hepatorenal insufficiency. Based on the postoperative pathological findings, the 95 participants were divided into the metastasis group (*n* = 45) and nonmetastasis group (*n* = 46), and the intergroup differences in basic information and ultrasonographic features were analyzed. The clinical data, including the sex, age, tumor stage, and prognostic information, of all patients who underwent surgery for MTC, were collected, collated with the follow-up data, and then retrospectively analyzed. Postoperative histopathology was performed to confirm MTC metastasis. Informed consent was obtained from the patients for all serological tests and surgical interventions. The study was approved by the hospital ethics committee.

### 2.2. Sources of Transcriptome Data

In April 2021, we downloaded data from The Cancer Genome Atlas (TCGA) database (https://portal.gdc.cancer.gov/) on thyroid cancer mRNA-seq (thyroid cancer samples, *n* = 507) and clinical information on thyroid cancer patients [14]. Normal thyroid tissue mRNA-seq data (thyroid samples, *n* = 447) were downloaded from the Genotype-Tissue Expression (GTEx) database (https://www.gtexportal.org/) and used for the validation of the dataset.

### 2.3. Detection of Serum Calcitonin

Without any stimulation, a 4 mL fasting venous blood sample was preoperatively collected in a serum separator tube from the patient, left to stand for 30–60 min, and centrifuged at 3000 r/min for 10 min, and the supernatant (serum) was pipetted out for analysis. The specimen was determined to be free of hemolysis and lipemia. The serum calcitonin level was measured by chemiluminescence immunoassay (CLIA). The serum calcitonin level was detected using a Siemens IMMULITE^®^ 2000 fully automated chemiluminescence immunoassay detector with the specified reagents (detection limit 2–2000 pg/mL).

### 2.4. Instrumentation and Image Analysis

The patient was placed in a supine position with the neck hyperextended to fully expose the anterior cervical region. The thyroid gland and the LN in the neck region were fully scanned, and longitudinal and transverse views of the lesion were photographed. The ultrasonographic images of the included cases were analyzed by two attending radiologists who were blinded to the patient grouping and other details. Disagreements, if any, were resolved by consensus following a discussion between the two radiologists. Ultrasonographic images were analyzed to determine the infiltrating range, location, diameter, echo, nodule edge, and blood flow to the thyroid nodule [15].

### 2.5. Statistical Analysis

All data analyses in this study were performed using SPSS 24.0 and R package (version 3.6.1). The quantitative data were analyzed using the *t-*test. Numerical data were analyzed using the chi-square or Fisher's exact test, and the results are expressed as frequencies. Consistent with the results of earlier studies, least absolute shrinkage and selection operators (LASSO) and Cox regression were used for screening variables. Logistic regression analysis was used to screen for LN metastasis-related ultrasonographic features and to construct a nomogram model [16–18]. The model was evaluated by plotting the receiver operating characteristic (ROC) curves. A logistic regression model was used to screen for risk factors associated with MTC transfer, and a nomogram model was constructed using the rms package as previous researches [19–22]. Both internal and external validations were performed with the original data from the training and validation sets, and the ROC curves were plotted using the rms package. The clinical benefit was evaluated by decision curve analysis (DCA), and DCA curves were used to evaluate the clinical applicability of the prediction model. Based on the probability of each threshold in the clinical impact curve, the study population was subdivided into the high-risk and actual high-risk populations following the nomogram model, wherein a wider overlap indicated better predictive performance of the model. *P* < 0.05 was considered indicative of a statistically significant difference.

### 2.6. Gene Enrichment Analysis

The relevant pathways wherein *CALCA* was involved in MTC were predicted using GSEA software (version 4.1.0) [23]. The Kyoto Encyclopedia of Genes and Genomes (KEGG) pathway enrichment analysis was performed using GSEA software, and the study population was divided into the high- and low-calcitonin-expression groups based on the median *CALCA* expression. The annotated gene set (c2.cp.kegg.v7.2.symbols.gmt) was selected as the reference gene set, and 1000 genomic alignments were run for each analysis. *P* < 0.05 and a false discovery rate (FDR) < 0.25 indicated significant enrichment. Plots were generated with the R software packages “plyr,” “ggplot2,” “grid,” and “gridExtra.”

## 3. Results

### 3.1. Screening of Prognosis-Associated Factors in MTC

**Figure 1:**
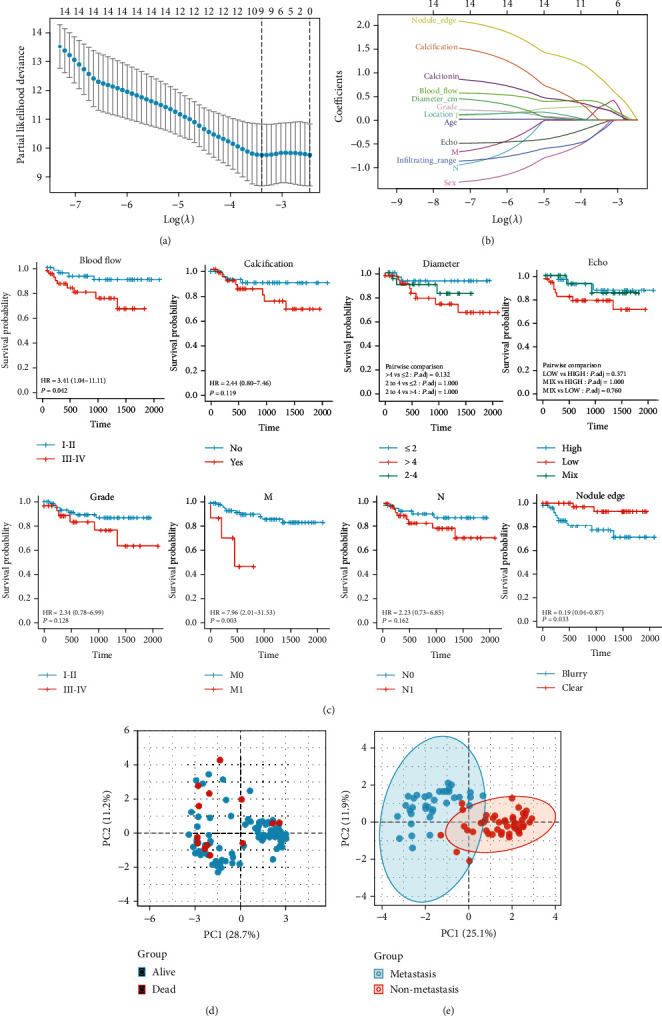
Screening for factors associated with prognosis and metastasis in medullary thyroid cancer (MTC). (a, b) Error-rate plot for 1000 cross-validations. (a) LASSO correlation coefficients for all factors of interest in this study. (b) Survival analysis based on the blood flow, calcification, diameter, echo, grade, M, N, and nodule edge as factors that are associated with MTC prognosis. (c, d) Principal component analysis was used to assess the ability of these data to stratify the prognosis and risk of metastasis in patients with MTC.

A total of 95 patients (age, mean ± SD, 56 ± 14.85 years) with MTC were included in this study; among them, 50 (52.63%) were male and 45 (47.37%) were female. There was no statistically significant intergroup difference between the death and survival groups for the age, metastasis, sex, tumor TNM grade, location, largest diameter, ultrasound echo, nodule edge status, presence/absence of calcification, blood flow, and calcitonin levels (*P* > 0.05). The tumor grade and range of infiltration were statistically significant (*P* < 0.01; Table 1). However, the age, tumor location, ultrasound echo, and nodule edge status of the participants did not differ statistically between the nonmetastasis and metastasis groups (*P* > 0.05). The infiltrating range, diameter, presence/absence of calcification, blood flow, preoperative calcitonin level, and MTC primary foci visualized on ultrasound differed significantly between the nonmetastasis and metastasis groups (*P* < 0.05; Table 2). The preoperative calcitonin level was significantly higher in the metastasis group, suggesting that the preoperative calcitonin level could help ascertain the possibility of metastasis in MTC. LASSO regression analysis showed that the factors that were associated with MTC prognosis included calcitonin, blood flow, calcification, diameter, echo, grade, M, N, and the nodule edge (Figures 1(a) and 1(b)). The results of the survival analysis suggested that blood flow, distant metastases, and nodule edge were significantly associated with the prognosis of MTC patients (Figure 1(c)). Univariate and multifactorial Cox analyses were used to assess the potential mechanisms that affect the prognosis of MTC patients (Table 3). Similar to the results of the LASSO regression analysis, the factors associated with MTC prognosis were identified as calcitonin, blood flow, calcification, diameter, echo, grade, M, N, and nodule edge (Table 3). We used principal component analysis (PCA) to assess the stratification of the prognosis and risk of metastasis in MTC patients based on these data (Figures 1(d) and 1(e)). The data set showed better performance in assessing the risk of metastasis and poor performance in assessing prognosis in MTC patients.

### 3.2. Screening for Prognostic and Metastasis Correlates of Medullary Thyroid Cancer (MTC)

**Figure 2:**
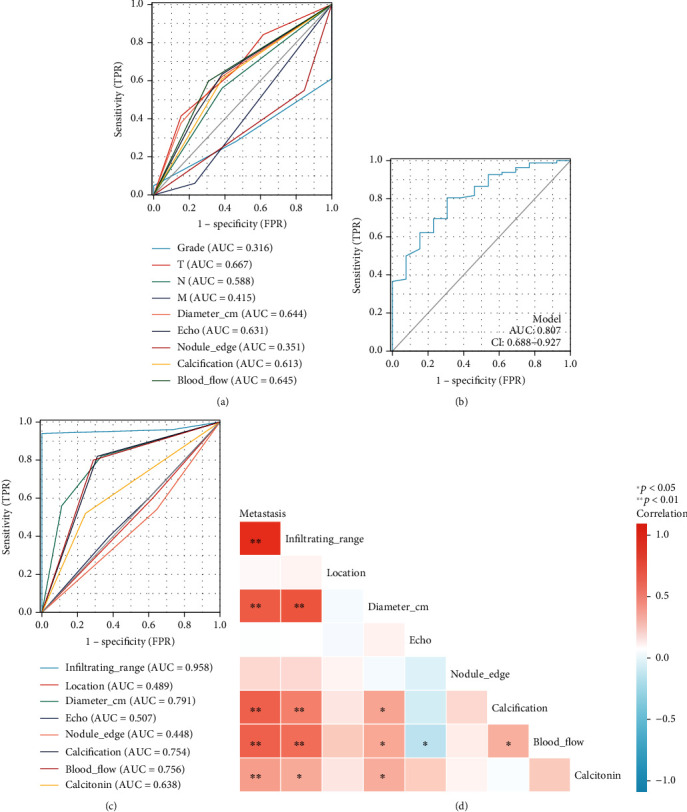
Screening for prognostic and metastasis correlates of medullary thyroid cancer (MTC). (a) Survival-related receiver operating characteristic (ROC) curves for factors associated with MTC prognosis; (b) an integrated analysis of survival-related ROC curves with factors that are associated with MTC prognosis; (c) ROC curve of factors associated with MTC metastasis risk; (d) correlational analysis of metastatic risk, infiltrating range, location, diameter, echo, nodule edge, calibration, blood flow, and calcitonin levels, wherein different colors indicate the correlation between the elements.

The ROC curve suggested that *T* (area under the curve [*AUC*] = 0.667), diameter (AUC = 0.644), echo (AUC = 0.631), calcification (AUC = 0.613), and blood flow (AUC = 0.645) could help predict the prognosis in MTC patients (Figure 2(a)). The integration of the results of the ROC curve suggested that these factors predicted the prognosis of MTC patients (AUC = 0.807, 95% CI 0.688–0.927; Figure 2(b)). The ROC curve suggested that infiltrating range (AUC = 0.958), diameter (AUC = 0.791) on ultrasonographic imaging, calcification (AUC = 0.754), and blood flow (AUC = 0.756) could predict the risk of MTC metastasis (Figure 2(c)). The serum calcitonin level was associated with MTC metastasis (AUC = 0.638; Figure 2(c)). Moreover, the results of the correlation analysis suggested that the risk of metastasis was significantly associated with the infiltrating range (AUC = 0.958), diameter (AUC = 0.791), calcification (AUC = 0.754), blood flow (AUC = 0.756), and serum calcitonin level (*P* < 0.01; Figure 2(d)).

### 3.3. Development and Validation of Predictive Models for Lymphatic Metastasis

**Figure 3:**
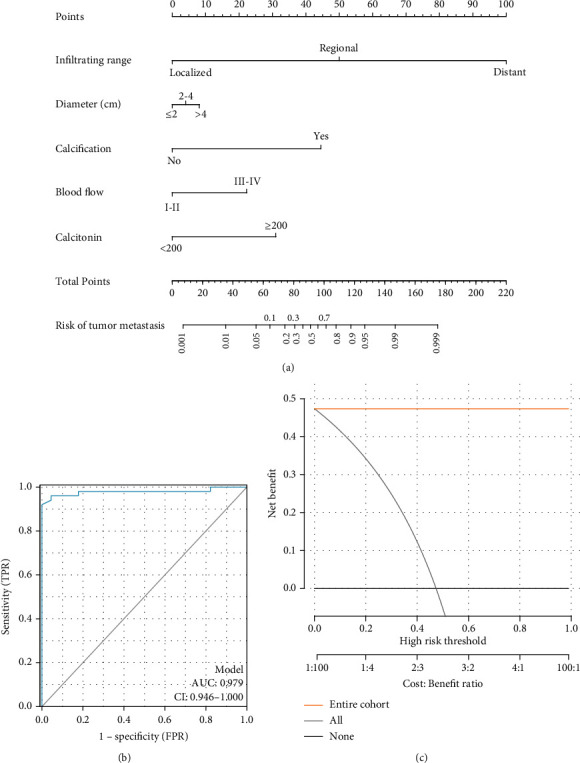
Construction of a nomogram prediction model for determining the risk of metastasis in medullary thyroid cancer (MTC) patients. (a) A prediction model comprising the infiltrating range, diameter, calcification, blood flow, and calcitonin level was constructed; (b) the receiver operating characteristic (ROC) curve reflects the predictive power of this nomogram prediction model; and (c) the decision curve analysis of the nomogram prediction model.

Using R software, factors, including infiltrating range, diameter, calcification, blood flow, and calcitonin level, with predictive power in determining the risk of metastasis were included to build a functional model and were plotted on a nomogram. The number or category of each of these factors was summed. Subsequently, the scores for each of these abovementioned factors were summed on the scale, and about the total score below, a straight line was drawn downwards; moreover, the intersection of the “risk of tumor metastasis” axis indicated the estimated survival time or probability at each time point (Figure 3(a) and Table 4). In the ROC curve, the nomogram prediction model estimated an AUC of 0.979 (95% confidence interval [CI] 0.946–1.000) for the risk of metastasis (Figure 3(b)). This suggests that the nomogram prediction model has good accuracy for determining the risk of metastasis in MTC, and the accuracy increased with the length of the postoperative period. A DCA of the nomogram prediction model which incorporated risk factors for metastasis showed that the nomogram prediction model has excellent clinical utility (Figure 3(c)).

### 3.4. Calcitonin-Related Functional Analysis

**Figure 4:**
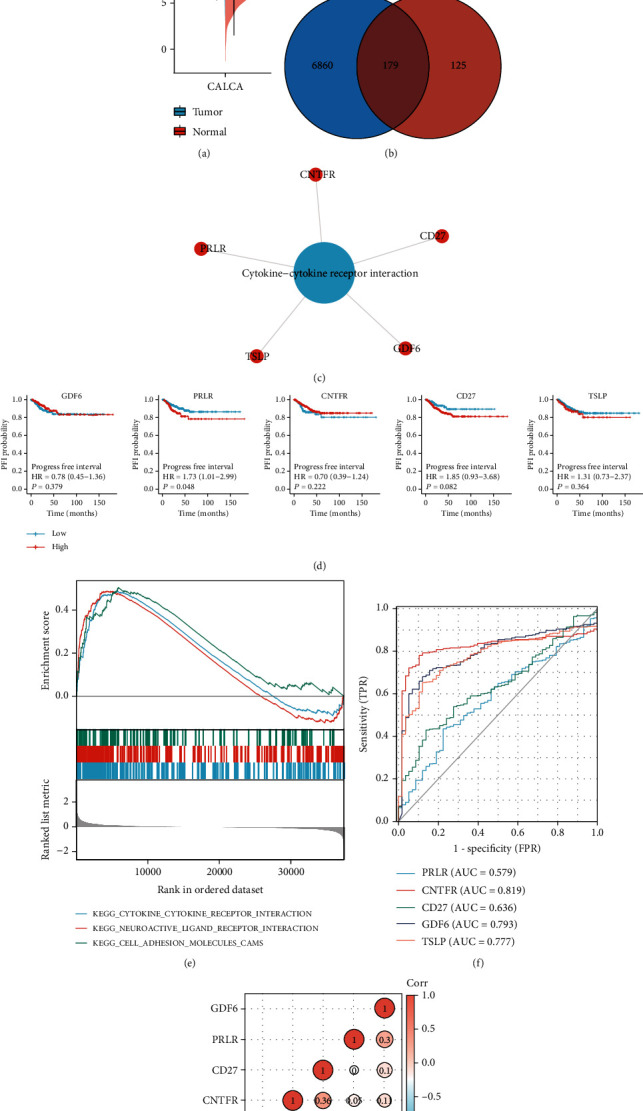
Analysis of calcitonin-related genes and functions. (a) Differential expression of *CALCA* in thyroid cancer tissues and normal tissues. (b) Venn diagrams of 7039 thyroid cancer-associated DEGs and 304 *CALCA*-associated DEGs. (c) 179 crossover genes were significantly enriched in the cytokine–cytokine receptor interaction pathway. (d) Survival analysis of five cytokine–cytokine receptor interaction pathway-related genes in thyroid cancer; (e) GSEA: KEGG pathways that are associated with differential *CALCA* expression; and (f) survival AUC curves derived from five cytokine–cytokine receptor interaction pathway-related genes in thyroid cancer.

Based on an integrated analysis of the TCGA and GTEx databases, we assessed the differential expression of the calcitonin mRNA *CALCA* in thyroid cancer tissues as well as in normal tissues. *CALCA* expression was significantly higher in thyroid cancer tissues (Figure 4(a) and Table 5). A Venn diagram that comprised 7039 thyroid cancer prognosis-related DEGs and 304 *CALCA*-related DEGs revealed 179 thyroid cancer prognosis-related genes that were associated with differences in *CALCA*-related expression (Figure 4(b)). Enrichment analysis of these 179 genes revealed that *CNTFR*, *PRLR*, *TSLP*, *GDF6*, and *CD27* were significantly enriched in the cytokine–cytokine receptor interaction pathway (Figure 4(c)).  Figure 4(d) shows the outcome of the survival analysis of five cytokine–cytokine receptor interaction pathway-related genes in thyroid cancer. High *PRLR* expression was significantly associated with poor prognosis in MTC. The results of GSEA suggested that high *CALCA* expression was significantly associated with the cytokine–cytokine receptor interaction pathway, neuroactive ligand-receptor interaction pathway, and cell-adhesion molecules. The ROC analysis indicated that the cytokine–cytokine receptor interaction pathway-related genes *CNTFR* (AUC = 0.819), *CD27* (AUC = 0.636), *GDF6* (AUC = 0.793), and *TSLP* (AUC = 0.777) were predictive of the MTC metastasis risk (Figure 4(f)).

## 4. Discussion

In this study, we assessed the correlation between the patient's age, sex, preoperative calcitonin levels, and multiple ultrasonographic signs and the development of lymphatic metastasis in MTC patients. Based on the correlation, we developed a nomogram model to visualize the risk factors that are associated with the lymphatic metastasis of MTC. The differential expression of calcitonin mRNA *CALCA* in thyroid cancer tissues and normal tissues and the expression of the associated genes and pathways were assessed based on data obtained from the databases.

Preoperative calcitonin testing, combined with ultrasound-based characterization of the primary foci of MTC, can facilitate the preoperative prediction of lymphatic metastasis and thereby improve the surgeons' understanding of MTC progression. We emphasize the key role of preoperative serum calcitonin levels and ultrasonographic evaluation of MTC primary foci in the assessment of the patient's risk of lymphatic metastasis to enable a risk-based selection of an appropriate surgical approach to achieve a good prognosis for the patient.

The results of this study indicated that age was unassociated with lymphatic metastasis, whereas sex was associated with LN metastasis; specifically, male MTC patients were more likely to develop cervical LN metastasis of MTC. Due to the high sensitivity and specificity in suggesting the presence of MTC, serum calcitonin levels have been used as a common thyroid serum tumor marker in the clinical diagnosis of MTC [24, 25]. The preoperative calcitonin level was associated with the risk of lymphatic metastasis in MTC. According to our study, the preoperative calcitonin levels were significantly higher in the metastasis group than in the nonmetastasis group, which is consistent with the results of previous studies [8]. However, an increase in calcitonin levels is not unique to MTC, and false-positive results can be caused by hypercalcemia, hypergastrinemia, differentiated thyroid cancer, goiter, and chronic autoimmune thyroiditis [26].

Among the multiple ultrasonographic features of MTC, this study found that the infiltrating range, diameter, presence of calcification, and blood flow of the primary foci of MTC differed significantly between the nonmetastasis and metastasis groups and could help predict the risk of metastasis to some extent. The thyroid gland is a highly vascularized endocrine organ, and the growth of a thyroid nodule mainly depends on internal vascularity. Therefore, the size of the nodules can reflect tumor growth to some extent. Large nodules indicate faster proliferation and a higher degree of tumor cell infiltration [27]. Moreover, nodules that protrude into the thyroid envelope may come into closer contact with the surrounding lymphatic vessels, thereby increasing the risk of cervical LN metastasis. Previous studies have shown that perineural infiltration is an independent predictor of LN metastasis to the central and lateral cervical regions in patients with MTC [28]. Due to the rich lymphatic network of the thyroid gland, the psammoma body in the tumor can be released into local tissues as the tumor cells are transferred to the extranodal glands or cervical lymph nodes via hematologic or lymphatic transport; therefore, microcalcifications can be detected when metastases appear in early stage MTC [8, 29, 30]. In addition, the faster growth of tumor cells and proliferation of fibrous tissue, which results in calcium salt deposition, may be responsible for microcalcification [8, 31]. The ROC curve indicated that *T*, diameter, echo, calcification, and blood flow could predict the prognosis of MTC, and this conclusion was consistent with the findings of Zhu and Xu [32]. In this study, a nomogram prediction model was developed based on five factors that were screened by ROC curve and were capable of predicting the risk of metastasis. The ROC curve revealed an AUC value of 0.979 (95% CI 0.946–1.000) for the prediction model, indicating that the model has good accuracy. The risk of lymphatic metastasis in MTC can be predicted clinically by summing the scores of each risk factor, thus enhancing the screening and management of controllable factors. This prediction model indicates that clinical staff should pay close attention to MTC patients with multiple risk factors to improve the preoperative examination and diagnosis as much as possible and to accurately assess the metastasis site and extent of resection necessary to improve surgical outcomes.

Our analysis of data from the TCGA and GTEx databases showed a significantly higher *CALCA* mRNA expression in thyroid cancer tissues, which is consistent with the results of a study by Camacho et al. [33]. Among the thyroid cancer, prognosis-related genes associated with differential *CALCA* expression, *CNTFR*, *PRLR*, *TSLP*, *GDF6*, and *CD27* were significantly enriched in the cytokine–cytokine receptor interaction pathway, and high *CALCA* expression was significantly associated with the cytokine–cytokine receptor interaction pathway, neuroactive ligand-receptor interaction pathway, and cell-adhesion molecules, which suggests that these pathways may be involved in the development of thyroid cancer and could affect the prognosis of MTC patients.

The use of preoperative basal calcitonin levels in combination with ultrasonographic diagnostic features can potentially benefit patients by enabling an accurate preoperative noninvasive diagnosis of MTC and determination of the risk of MTC metastasis. However, there are certain limitations of this study that need be mentioned: (1) the retrospective study design confers a risk of selection bias; (2) concerning the interpretation of ultrasonographic images, there was interobserver agreement bias; when the tumor size is too small, the internal blood flow characteristics cannot be determined, which leads to a higher rate of misdiagnosis or underdiagnosis; moreover, the complexity of the physiological structure of the thyroid gland may affect the echo findings of the tumor, thereby resulting in an overlapping of the resultant images; and (3) this study was limited by a small sample and limited data range and lacked information from multicenter, randomized large samples; thus, there is a need to expand the range of the sample and to increase the sample size for an in-depth study.

In summary, the preoperative serum calcitonin level, in combination with ultrasonographic findings, improves the preoperative risk prediction of MTC metastasis. Thus, this combination could be used as a preoperative noninvasive and accurate diagnostic method for predicting MTC metastasis. An improved surgeons' awareness of MTC is essential to improve the clinical prognosis of MTC patients, and this emphasizes the need for preoperative determination of serum calcitonin levels and ultrasonographic findings to facilitate the selection of a standardized surgical approach. There are many risk factors for metastasis in MTC patients, and the establishment of the related nomogram prediction model is beneficial for clinical screening of groups that are at high risk for metastasis to formulate relevant prevention and control measures, which has guiding significance for further optimizing the quality of surgery and improving the patients' prognosis. Thus, the novel method evaluated in this study has high clinical applicability and is worthy of further promotion and use.

## 5. Conclusion

A nomogram based on preoperative serum calcitonin levels and the infiltrating range, diameter, calcification, blood flow, and other ultrasonographic characteristics of MTC can be used as an intuitive and noninvasive quantitative tool to predict the risk of lymphatic metastasis in MTC and facilitate the individualization of the preoperative LN surgical debulking strategy.

## Figures and Tables

**Table 1:** The relationship between ultrasound characteristics, clinicopathological characteristics, and survival status.

Characteristic	Dead (*n* = 13)	Alive (*n* = 82)	*p*
Survival (months), median (IQR)	10 (7, 19)	36 (14.5, 52.75)	0.005
Age, median (IQR)	61 (54, 69)	57.5 (46.5, 67)	0.242
Group, *n* (%)			0.161
Metastasis	9 (9.5%)	36 (37.9%)	
Nonmetastasis	4 (4.2%)	46 (48.4%)	
Sex, *n* (%)			0.694
Female	5 (5.3%)	40 (42.1%)	
Male	8 (8.4%)	42 (44.2%)	
Grade, *n* (%)			0.011
I	0 (0%)	32 (33.7%)	
II	7 (7.4%)	27 (28.4%)	
III	6 (6.3%)	19 (20%)	
IV	0 (0%)	4 (4.2%)	
T, *n* (%)			0.141
T1	2 (2.1%)	34 (35.8%)	
T2	4 (4.2%)	20 (21.1%)	
T3	2 (2.1%)	15 (15.8%)	
T4	5 (5.3%)	13 (13.7%)	
N, *n* (%)			0.376
N0	5 (5.3%)	46 (48.4%)	
N1	8 (8.4%)	36 (37.9%)	
M, *n* (%)			0.075
M0	10 (10.5%)	77 (81.1%)	
M1	3 (3.2%)	5 (5.3%)	
Infiltrating range, *n* (%)			0.020
Distant	5 (5.3%)	9 (9.5%)	
Localized	3 (3.2%)	44 (46.3%)	
Regional	5 (5.3%)	29 (30.5%)	
Location, *n* (%)			0.730
Left	9 (9.5%)	49 (51.6%)	
Right	4 (4.2%)	33 (34.7%)	
Diameter (cm), *n* (%)			0.191
>4	8 (8.4%)	31 (32.6%)	
2–4	3 (3.2%)	20 (21.1%)	
≤2	2 (2.1%)	31 (32.6%)	
Echo, *n* (%)			0.269
High	3 (3.2%)	34 (35.8%)	
Low	8 (8.4%)	30 (31.6%)	
Mix	2 (2.1%)	18 (18.9%)	
Nodule edge, *n* (%)			0.085
Blurry	11 (11.6%)	45 (47.4%)	
Clear	2 (2.1%)	37 (38.9%)	
Calcification, *n* (%)			0.221
No	5 (5.3%)	50 (52.6%)	
Yes	8 (8.4%)	32 (33.7%)	
Blood flow, *n* (%)			0.098
I–II	4 (4.2%)	49 (51.6%)	
III–IV	9 (9.5%)	33 (34.7%)	
Calcitonin, *n* (%)			0.730
<200	4 (4.2%)	33 (34.7%)	
≥200	9 (9.5%)	49 (51.6%)	

**Table 2:** The relationship between ultrasound characteristics, clinicopathological characteristics, and metastasis status.

Characteristic	Nonmetastasis	Metastasis	*P*
Status, *n* (%)			0.161
Alive	46 (48.4%)	36 (37.9%)	
Dead	4 (4.2%)	9 (9.5%)	
Age, median (IQR)	62 (46.5, 68)	57 (49, 64)	0.410
Sex, *n* (%)			0.001
Female	32 (33.7%)	13 (13.7%)	
Male	18 (18.9%)	32 (33.7%)	
Grade, *n* (%)			<0.001
I	25 (26.3%)	7 (7.4%)	
II	21 (22.1%)	13 (13.7%)	
III	4 (4.2%)	21 (22.1%)	
IV	0 (0%)	4 (4.2%)	
T, *n* (%)			<0.001
T1	29 (30.5%)	7 (7.4%)	
T2	12 (12.6%)	12 (12.6%)	
T3	7 (7.4%)	10 (10.5%)	
T4	2 (2.1%)	16 (16.8%)	
N, *n* (%)			<0.001
N0	50 (52.6%)	1 (1.1%)	
N1	0 (0%)	44 (46.3%)	
M, *n* (%)			0.002
M0	50 (52.6%)	37 (38.9%)	
M1	0 (0%)	8 (8.4%)	
Infiltrating range, *n* (%)			<0.001
Distant	2 (2.1%)	12 (12.6%)	
Localized	47 (49.5%)	0 (0%)	
Regional	1 (1.1%)	33 (34.7%)	
Location, *n* (%)			0.991
Left	30 (31.6%)	28 (29.5%)	
Right	20 (21.1%)	17 (17.9%)	
Diameter (cm), *n* (%)			<0.001
>4	9 (9.5%)	30 (31.6%)	
≤2	28 (29.5%)	5 (5.3%)	
2 to 4	13 (13.7%)	10 (10.5%)	
Echo, *n* (%)			0.958
High	20 (21.1%)	17 (17.9%)	
Low	20 (21.1%)	18 (18.9%)	
Mix	10 (10.5%)	10 (10.5%)	
Nodule edge, *n* (%)			0.410
Blurry	27 (28.4%)	29 (30.5%)	
Clear	23 (24.2%)	16 (16.8%)	
Calcification, *n* (%)			<0.001
No	41 (43.2%)	14 (14.7%)	
Yes	9 (9.5%)	31 (32.6%)	
Blood flow, *n* (%)			<0.001
I–II	40 (42.1%)	13 (13.7%)	
III–IV	10 (10.5%)	32 (33.7%)	
Calcitonin, *n* (%)			0.011
<200	26 (27.4%)	11 (11.6%)	
≥200	24 (25.3%)	34 (35.8%)	

**Table 3:** Univariate and multifactorial COX analyses of the risk of death in patients with medullary thyroid cancer.

Characteristics	Univariate analysis		Multivariate analysis	
	Hazard ratio (95% CI)	*P* value	Hazard ratio (95% CI)	*P* value
Sex	1.572 (0.514–4.810)	0.428		
Grade	1.806 (1.011–3.226)	0.046	1.130 (0.423–3.013)	0.808
T	1.749 (1.085–2.819)	0.022	2.033 (0.614–6.734)	0.246
N	2.230 (0.725–6.853)	0.162	0.222 (0.032–1.519)	0.125
M	7.957 (2.008–31.531)	0.003	7.942 (1.476–42.721)	0.016
Infiltrating range	1.452 (0.816–2.584)	0.204		
Location	1.541 (0.475–5.007)	0.472		
Diameter (cm)	2.010 (0.984–4.105)	0.055	0.973 (0.174–5.433)	0.975
Echo	0.586 (0.298–1.153)	0.122	0.744 (0.325–1.706)	0.485
Nodule edge	5.179 (1.145–23.432)	0.033	5.136 (0.949–27.787)	0.057
Calcification	2.438 (0.796–7.463)	0.119	1.858 (0.465–7.431)	0.381
Blood flow	3.405 (1.044–11.111)	0.042	2.794 (0.712–10.959)	0.141
Calcitonin	1.506 (0.463–4.895)	0.496		

**Table 4:** Model parameters for ultrasound combined with calcitonin to predict metastasis in medullary thyroid cancer.

Items	Regression coefficient	OR	OR (95% CI)	*P* value
Infiltrating range	4.117	61.363	6.233~604.133	*P* <0.001
Diameter (cm)	0.334	1.396	0.327~5.960	0.652
Calcification	3.666	39.077	2.544~600.229	0.009
Blood flow	1.833	6.255	0.817~47.896	0.078
Calcitonin	2.554	12.852	0.772~213.857	0.075
Intercept	−7.495	0.001	0.000~0.054	0.001

**Table 5:** Correlation of *CALCA* expression with clinical characteristics.

Characteristic	Low expression of *CALCA* (*n* = 251)	High expression of *CALCA* (*n* = 251)	*P* value
Age, *mean* ± *SD*	48.16 ± 15.84	46.53 ± 15.82	0.248
T stage, *n* (%)			0.035
T1	64 (12.8%)	79 (15.8%)	
T2	89 (17.8%)	75 (15%)	
T3	80 (16%)	90 (18%)	
T4	17 (3.4%)	6 (1.2%)	
N stage, *n* (%)			0.455
N0	121 (26.8%)	108 (23.9%)	
N1	109 (24.1%)	114 (25.2%)	
M stage, *n* (%)			0.532
M0	142 (48.8%)	140 (48.1%)	
M1	6 (2.1%)	3 (1%)	
Pathologic stage, *n* (%)			0.514
Stage I	138 (27.6%)	143 (28.6%)	
Stage II	23 (4.6%)	29 (5.8%)	
Stage III	57 (11.4%)	55 (11%)	
Stage IV	32 (6.4%)	23 (4.6%)	
Gender, *n* (%)			1.000
Female	184 (36.7%)	183 (36.5%)	
Male	67 (13.3%)	68 (13.5%)	
Race, *n* (%)			0.860
Asian	25 (6.1%)	26 (6.3%)	
Black or African	15 (3.7%)	12 (2.9%)	
American white	170 (41.5%)	162 (39.5%)	

## Data Availability

Thyroid cancer mRNA-seq data (thyroid cancer samples: *n* = 507) and clinical information of the corresponding patients were downloaded from the TCGA database. Normal thyroid tissue mRNA-seq data (thyroid samples: *n* = 447) were downloaded from the GTEx database. Clinical data can be obtained by contacting the corresponding author.

## Abbreviations

CLIA: Chemiluminescence immunoassay
CALCA: Calcitonin
DCA: Decision curve analysis
LASSO: Least absolute shrinkage and selection operators
LN: Lymph node
MTC: Medullary thyroid carcinoma
PCA: Principal component analysis.
